# Vitiligo Appearing after Oral Isotretinoin Therapy for Acne

**DOI:** 10.1155/2018/3697260

**Published:** 2018-07-12

**Authors:** Amal A. Kokandi

**Affiliations:** Department of Dermatology, Faculty of Medicine, King Abdulaziz University, Jeddah, Saudi Arabia

## Abstract

Isotretinoin is an effective treatment for severe and scarring acne. In this report, we describe a case developing vitiligo after isotretinoin therapy for scarring acne. It is not known whether this was a coincidence or might be precipitated by the treatment.

## 1. Introduction

Isotretinoin is an effective therapy for severe acne. It has the best influence on the health-related quality of life in acne patients [1]. Some side effects of isotretinoin are well known and predictable such as chelitis and xerosis. Other side effects are known but less common such as hyperlipidemia. It is a known teratogenic medication and is contraindicated in pregnancy. Several other side effects are reported but less commonly [2]. Depressive symptoms and suicidal ideation are other issues for the use of isotretinoin [3].

Vitiligo is an autoimmune disease of the skin and is thought to be of multifactorial causation [4, 5]. Vitiligo patients have high prevalence of other autoimmune diseases [6]. Thyroid function tests and thyroid autoantibodies might be used for screening for vitiligo patients [7].

Here we report a case of vitiligo appearing after isotretinoin therapy. It is unknown whether this was a side effect of oral isotretinoin therapy or a coincidence.

## 2. Case Presentation

A 21-year-old female presented to the dermatology clinic with severe facial acne with some scars. Severity of acne was graded as 4 on IGA scale (investigator global assessment of acne) which is accepted by American FDA [8]. She has used topical treatments including topical retinoids (Tretinoin and Adapalene creams) for several months with no satisfactory results. On presentation, she did not have any other complaints and was not on any systemic treatments. Her weight was 45 kg.

After initial laboratory works (lipid profile and liver enzymes) which were in the normal range, she was started on 20 mg isotretinoin. She was maintained on 20 mg (0.5 mg/kg) for 6 months. She had mild chelitis and skin dryness and complained of mild hair fall. Repeated liver enzymes and lipid profile after one month and 4 months were within normal range. Her acne has cleared completely.

She stopped the treatment because of inability to attend the clinic for few weeks.

**Figure 1:**
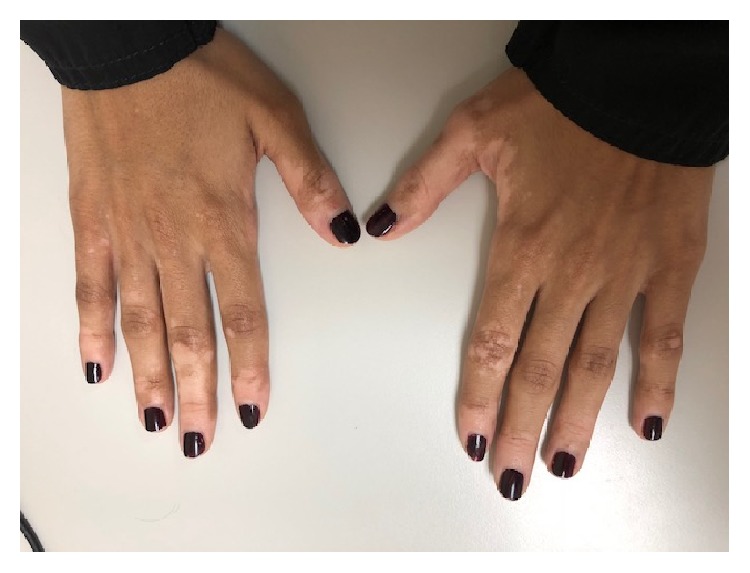
Vitiligo lesions in the hands.

After 2 months of stopping isotretinoin, she noticed a single whitish patch on her nose. She is fair-skinned, so the lesions were not apparent except on tanning after sun exposure. Antifungal treatment was used for few weeks topically with no improvement as it was thought to be pityriasis versicolor. Then the lesion began to expand, and new lesions appeared around mouth, cheeks, and right ankle area. Hand lesions appeared as well (Figure 1). On Wood's light examination, the patches were revealed to be depigmented. The pattern of acrofacial vitiligo is noted [5].

Thyroid function test initially showed low TSH, 0.177 uIU/L (normal range: 0.27-4.2), and normal levels of free T3, 6.11 pmol/L (2.8- 7), and free T4, 15.7 pmol/L (12-22). Three months later, TSH was high, 9.61 uIU, and normal free T3 (4.7 pmol/L) and free T4 (12.2 pmol/L) and thyroid antibodies were positive; thyroid peroxidase antibodies were 157.59 IU/mL (normal range: 0-5.6) and thyroid thyroglobulin antibodies were 66.09 IU/mL (normal range: 0-4.11). She was started on thyroxine and followed up at the medical clinic. Vitamin D3 was low, 47.11 nmol/L (normal range: 75-250 nmol/L), and she was started on vitamin D supplement as well. She had no family history of vitiligo. There was a family history of diabetes, hypertension, and systemic lupus erythematosus (SLE) and her auntie died from renal complication of SLE.

She was started on Tacrolimus 0.1% cream. Mild improvement was noted in some of lesions after 8 weeks. New lesions appeared again after another month. She stopped the topical treatment and opted to homeopathic treatment.

## 3. Discussion

In this report, we describe a case of vitiligo appearing for the first time after using oral isotretinoin for scarring acne. The most common side effects with oral isotretinoin therapy are skin dryness and chelitis. In a study of 1743 cases reviewing the side effects of isotretinoin, chelitis was reported to be the most common side effect followed by eczema and tiredness [9]. Of note, vitiligo was not reported in this study. Some rare complications have been reported like acute myocardial infarction linked with the increase in lipids [10], inflammatory bowel disease [11], and severe myopathy [12].

Vitiligo was reported around lips in a patient treated with isotretinoin owing to the chelitis as a Koebner phenomenon. This patient suffered from vitiligo prior to the initiation of isotretinoin [13]. Our case is different as she developed vitiligo lesions for the first time after isotretinoin therapy. One patient out of 50 developed vitiligo while on low-dose (20 mg/day for 3 months) isotretinoin therapy for acne [14], while larger-scale studies did not report vitiligo as a side effect (150 patients) [15].

Vitiligo is a multifactorial polygenic with incomplete penetrance inherited disease with a significant environmental influence. Autoimmune diseases, especially thyroid diseases, are very common association with vitiligo [16]. In vitro studies have shown that retinoids may have a proapoptotic effect on melanocytes [17]. Melanocytes express some retinoid receptors that are lost in melanoma compared to benign nevi [18], which could be a direct cause of vitiligo in this case. Additionally, retinoids might increase inflammatory cytokines via mast cells [19]. In this case, it is unknown whether oral isotretinoin acted here as a trigger to induce the disease via one of the pathways in a susceptible individual or it was a mere coincidence. Reporting similar cases if happening can help identify whether this is a true side effect of the medication.
